# The comparison of corneal higher-order aberration and surgically induced astigmatism between the clear corneal incision and the limbus tunnel incision of posterior chamber implantable collamer lens implantation

**DOI:** 10.1186/s12886-024-03311-1

**Published:** 2024-01-25

**Authors:** Ting-Ting Dan, Tai-Xiang Liu, Hong-yang Luo, Yi-Lu Liao, Zong-Ze Li

**Affiliations:** 1 Department of Ophthalmology, The Affiliated Hospital of Zunyi Medical University, Huichuan District, No. 149 Dalian Road, Zunyi, 563003 Guizhou Province China; 2 Guizhou Eye Hospital, Zunyi, 563000 Guizhou Province China; 3 Guizhou Provincial Branch of National Eye Disease Clinical Research Center, Zunyi, 563000 Guizhou Province China; 4 Special Key Laboratory of Ocular Diseases of Guizhou Province, Zunyi Medical University, Zunyi, 563000 Guizhou Province China

**Keywords:** Implantable collamer lens, Surgically induced astigmatism, Corneal aberration, Clear corneal incision, Limbus tunnel incision

## Abstract

**Background:**

This study aimed to compare the corneal high-order aberrations and surgically induced astigmatism between the clear corneal incision and limbus tunnel incision for posterior chamber implantable collamer lens (ICL/TICL) implantation.

**Methods:**

A total of 127 eyes from 73 myopic patients underwent ICL V4c implantation, with 70 eyes receiving clear corneal incisions and 57 eyes receiving limbus tunnel incisions. The anterior and back corneal surfaces were measured and the Root Mean Square of all activated aberrations (TRMS) was calculated, including higher-order aberration (HOA RMS), spherical aberration Z_4_^0^, coma coefficients (Coma RMS) Z_3_^−1^ Z_3_^1^, and surgically induced astigmatism (SIA). The measurements were taken preoperatively and postoperatively at 1 day, 1 week, and 1, 3, and 6 months. In this study, the corneal higher-order aberration was estimated as the Zernike coefficient calculated up to 5th order. The measurements were taken at a maximum diameter of 6.5 mm using Pentacam.

**Results:**

One week after the operation, the corneal back Z_3_^1^ of the clear corneal incision group was 0.06 ± 0.06, while the limbus tunnel incision group showed a measurement of 0.05 ± 0.06 (*p* = 0.031). The corneal back Z_4_^0^ of the clear corneal incision group was -0.02 ± 0.25, compared to -0.04 ± 0.21 in the limbus tunnel incision group (*p* = 0.01). One month after the operation, the corneal back SIA of the clear corneal incision group was 0.11 ± 0.11, compared to 0.08 ± 0.11of the limbus tunnel incision group (*p* = 0.013), the corneal total SIA of the clear corneal incision group was 0.33 ± 0.30, compared to 0.15 ± 0.16 in the limbus tunnel incision group (*p* = 0.004); the clear corneal incision group exhibited higher levels of back astigmatism and total SIA than the limbus tunnel incision in the post-operation one month period. During the 6- month post-operative follow-up period, no significant difference in Z_3_^1^, Z_4_^0^, and other HOA RMS data was observed between the two groups. The total SIA of the corneal incision group and the limbus tunnel incision group were 0.24 ± 0.14 and 0.33 ± 0.32, respectively (*p* = 0.393), showing no significant difference between the two groups 6 months after the operation.

**Conclusion:**

Our data showed no significant difference in the high-order aberration and SIA between clear corneal incision and limbus tunnel incision up to 6 months after ICL-V4c implantation.

## Background

The posterior chamber implantable collamer lens (ICL/TICL) is used for the correction of moderate to high myopia. This intraocular refractive surgery has been approved by the U.S. Food and Drug Administration since 2005 [1]. Comparative studies have reported that ICL provides superior spectacle-corrected visual acuity and refractive predictability and stability compared to keratoconus surgery. Additionally, ICL has a lower risk of retinal detachment compared to refractive lens exchange [2]. This technique results in stable visual quality and safety after 3 to 10 years of follow-up [3–5]. Stability is maintained due to minimal corneal damage and a structure that closely resembles the normal corneal structure in patients with high-risk factors for keratoconus [6, 7].

Corneal refractive surgery is one of the most popular surgical methods for correcting myopia, including RPR LASIK and Smile [8]. However, ocular aberration and changes in visual quality have been reported in both surgical approaches. Corneal aberration influences optics quality and is the primary cause of intraocular refractive aberration [9].

Precise measurements and a carefully planned procedure are essential to optimize visual quality. The corneal incision may be a factor that needs to be considered for more accurate calculations of the refractive index. IOL calculations for cataract surgery take into account the position of the surgical incision, its width, the potential astigmatism of the surgical site, and the effect of aberration changes on the patient's visual quality [10]. Nevertheless, current ICL surgery calculations do not consider these parameters, and the methods have been based on cataract surgery. Corneal incisions flatten the perpendicular corneal meridian, which may lead to surgically induced astigmatism (SIA). The magnitude of SIA depends on factors such as the size, location, and number of the incision [11–13]. Multiple factors, such as corneal distortion, turbulence in the irrigation solution, mechanical trauma from instruments, contact with nuclear fragments and intraocular lens, and the presence of free oxygen radicals, all contribute to corneal damage during cataract surgery [14]. Unlike cataract surgery, ICL surgery involves fewer incisions and a shorter operative time, while the cornea is not exposed to super-generated energy. These differences may potentially result in varied aberrations and changes in astigmatism. Therefore, this study aimed to compare the corneal high-order aberrations and surgically induced astigmatism (SIA) between the clear corneal incision and limbus tunnel incision for posterior chamber implantable collamer lens (ICL/TICL) implantation at different time points.

## Methods

### Patients

A total of 127 eyes from 73 myopic patients (aged 29.3 ± 6.6 years, 16 males and 57 females) who underwent ICL V4c implantation at the Myopic Center, Zunyi Medical University Affiliated Hospital from December 2017 to January 2021 were enrolled retrospectively. A clear incision was performed on 70 eyes, and a limbus tunnel incision was carried out on 57 eyes. Informed consent was obtained from all of the patients prior to the study, and the protocol adhered to the principles of the Declaration of Helsinki. This retrospective study was approved by Zunyi Medical University’s Institutional Ethics Committee. A corneal endothelial cell count of more than 2000/mm^2^ was indicative of the absence of viral or bacterial infection. The exclusion criteria comprised a history of previous ocular surgery, surgical complications, any corneal or macular pathology, refractive instability, poor fixation, etc. Cases involving severe dry eyes were also excluded.

### Surgical techniques

**Fig. 1:**
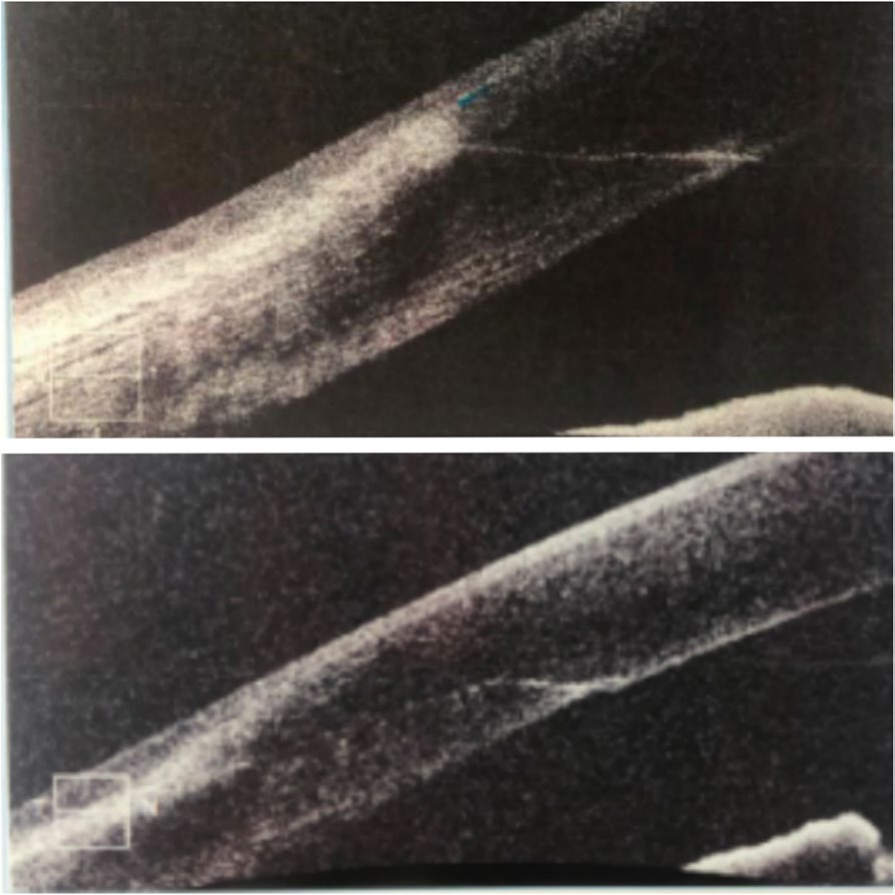
The OCT images of the clear corneal incision and the limbus tunnel incision

All surgeries were performed by the same experienced surgeon, Dr. LTX. The patients were randomly selected and divided into two groups based on the corneal incision performed. Group A received a clear corneal incision of approximately 0.5 mm in front of the corneal scleral rim. Group B received a limbus tunnel incision of 1.0 mm posterior to the corneal limbus. All ICL surgery was performed through a 3.0 mm corneal incision at 11:00 o’clock (Fig. 1). The corneal incision was hydrated at the end of the surgery without any auxiliary incision.

Pupils were sufficiently dilated with tropicamide (Santen Pharmaceutical Co., Ltd., Osaka, Japan) before the surgery. Topical anesthesia was achieved with proparacaine (proparacaine hydrochloride, Alcon), and the foldable V4-ICL was inserted into the posterior chamber through a 2.8 mm corneal or limbus incision using a specialized injector. The ICL was placed in the ciliary sulcus and adjusted to a suitable angle by using a gauge. Afterward, the remaining viscoelastic agent in the anterior chamber was completely removed using irrigation/aspiration (I/A) with a balanced saline solution. Subsequently, the patients were administered a steroidal agent (0.1% fluorometholone, Santen, Osaka, Japan), an antibiotic (0.3% levofloxacin, Santen, Osaka, Japan), an NSAID (Pranoprofen, Senju Pharmaceutical, Japan), and sodium hyaluronate (1.7% sodium hyaluronate; Bausch & Lomb, China), which were gradually reduced over the course a month.

### Ophthalmologic measurements

**Fig. 2:**
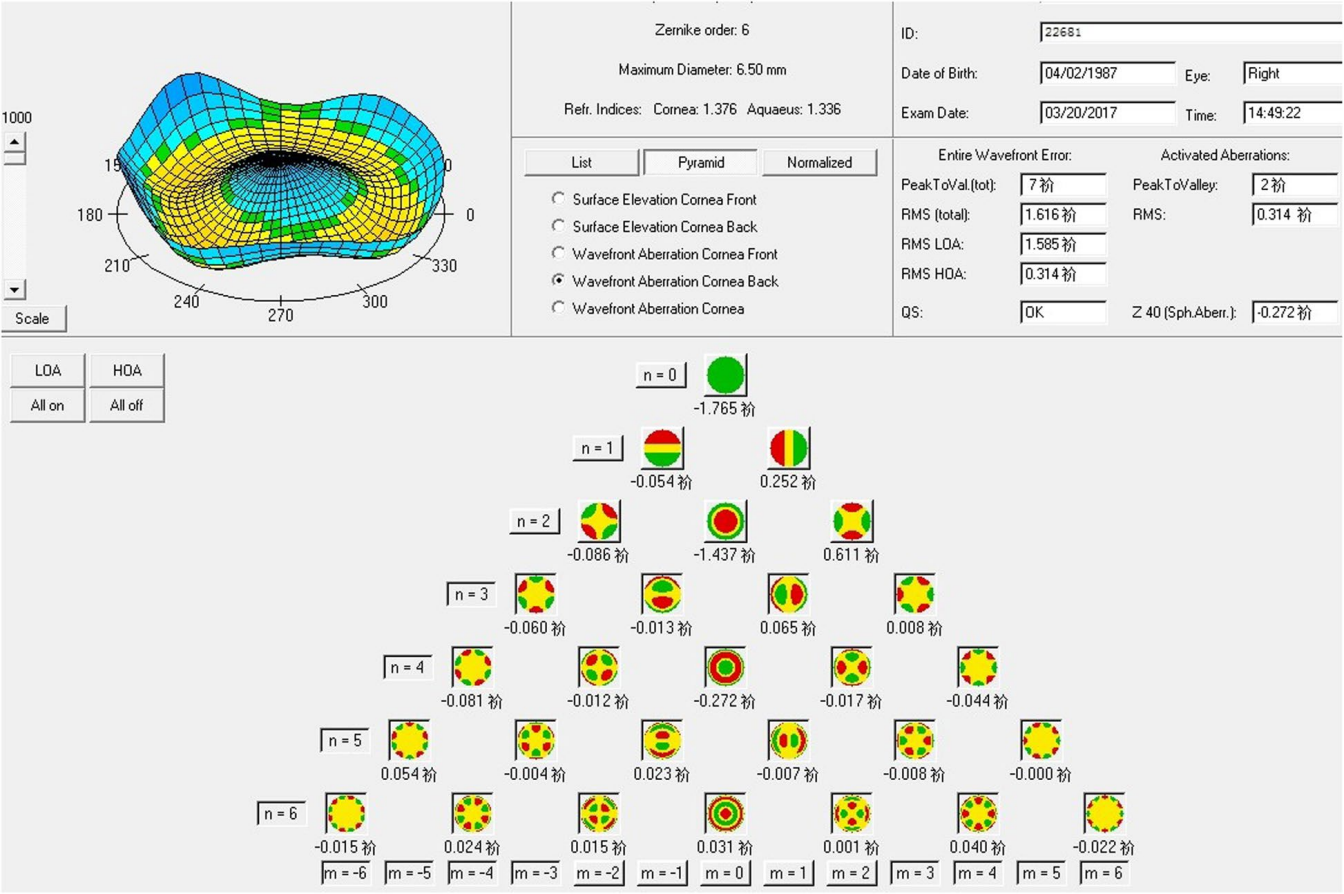
The corneal Higher-order aberration post and pre-surgery Zernike coefficients were measured by the Scheimpflug analysis system Pentacam

Ophthalmologic measurements were evaluated preoperatively and postoperatively at 1 day, 1 week, and 1, 3, and 6 months. The following measurements were taken: uncorrected distance visual acuity (UDVA), manifest refraction, best corrected visual acuity manifest refraction (CDVA), endothelial cell count (ECC; Topcon SP-2000P; Topcon, Tokyo, Japan), intraocular pressure (IOP) using a TX-10 non-contact tonometer (Canon, Japan), axial length, and anterior chamber depth using IOLMaster-500 (Carl Zeiss Meditec AG, Germany). The corneal higher-order aberration Zernike coefficients were measured before and after the procedure using the Scheimpflug analysis system Pentacam HR (Oculus®, Wetzlar, Germany). The Zernike coefficient was calculated up to the 5th order with a maximum diameter of 6.5 mm (Fig. 2). The wavefront aberration included the root mean square (RMS) of all HOA (HOA RMS), the total trefoil coefficients (TRMS), horizontal coma Z_3_^−1^, vertical coma Z_3_^1^, and the fourth-order spherical aberration Z_4_^0^ from the anterior aorneal surface, back aorneal surface, and total corneal.

### Statistical analysis

Data were expressed as mean ± SD. All the data were analyzed using SPSS Statistics 19.0 (SPSS Inc., Chicago, US), and the Kolmogorov–Smirnov test was used to confirm data normality. Independent t-tests were used for continuous variables, and Wilcoxon tests were used to compare continuous variables that did not follow a normal distribution.

## Result

In this study, 127 eyes from 73 myopic patients were included (Table 1). The clear corneal incision group included 70 eyes and the limbus tunnel incision group included 57 eyes. The groups were appropriately matched in terms of age, sex, ocular axis length (AL), uncorrected distance visual acuity (UDVA), corrected visual acuity (CDVA), and spherical equivalent degree (SE). No significant difference was observed between the two groups in terms of subjection (Table 1).

**Table 1:** Preoperative and operative baseline characteristics of patients

					Preoperative			Postoperative 6-month		
Group	n	SexF/M	Age	AL	UDVA	CDVA	SE	UDVA	CDVA	SE
CCI	70.00	46/24	24.60 ± 5.72	26.98 ± 1.08	1.37 ± 0.30	0.11 ± 0.22	-8.54 ± 2.94	-0.01 ± 0.13	-0.01 ± 0.22	0.10 ± 0.79
LTI	57	31/26	24.57 ± 4.70	26.40 ± 1.09	1.41 ± 0.27	0.06 ± 0.27	-7.98 ± 3.03	-0.02 ± 0.10	-0.01 ± 0.21	0.13 ± 0.66
P			0.306	0.460	0.43	0.15	0.819	0.17	0.336	0.754

*CCI* is the clear corneal incision group, *LTI* is the limbus tunnel incision group, *Sex F/M* is the number of female and male of patients, *Age* is the years of patients, *AL* is the ocular axis length, *UDVA* is the uncorrected distance visual acuity, *CDVA* is the best corrected visual acuity manifest refraction, *SE* is the spherical equivalent degree

### HOA of anterior corneal surface

The data collected one week after the operation showed no significant differences in terms of TMAS (*p* = 0.559); HOA (*p* = 0.328), Z_3_^−1^ (*p* = 0.328), Z_3_^1^ ( *p* = 0.786), and Z_4_^0^ (*p* = 0.175) between the clear corneal incision group and the limbus tunnel incision group. However, significant differences in TMAS (*p* = 0.001) and Z_3_^−1^ (*p* = 0.031) were found between the two groups 6 months after the operation; there was no significant difference between the two groups in terms of HOA (*p* = 0.716), Z_3_^1^ (*p* = 0.908), and Z_4_^0^(*p* = 0.954). Moreover, no significant differences were found between the two groups at other time points (all *p* > 0.05) (Table 2).

**Table 2:** The HOA on the anterior corneal surface of the two surgical incisions groups (mean ± SD)

Group	Time	n	TMAS	HOA	Z_3_^−1^	Z_3_^1^	Z_4_^0^
CCI	Preoperative	70	2.43 ± 0.75	0.54 ± 1.07	0.05 ± 0.16	0.06 ± 0.35	0.31 ± 0.19
	1Week		2.74 ± 0.88	0.77 ± 0.47	0.08 ± 0.33	0.11 ± 0.20	0.27 ± 0.12
	1Month		2.68 ± 0.79	0.63 ± 0.16	0.03 ± 0.13	0.48 ± 0.12	0.28 ± 0.27
	3Month		2.43 ± 0.81	0.67 ± 0.31	-0.01 ± 0.25	0.08 ± 0.19	0.30 ± 0.10
	6Month		2.53 ± 0.60	0.60 ± 0.16	0.12 ± 0.23	0.12 ± 0.21	0.32 ± 0.09
LTI	Preoperative	57	2.61 ± 1.07	0.60 ± 0.14	0.12 ± 0.25	0.09 ± 0.17	0.33 ± 0.12
	1Week		2.99 ± 1.50	0.74 ± 0.54	0.03 ± 0.75	0.12 ± 0.19	0.30 ± 0.14
	1Month		2.66 ± 1.28	0.97 ± 0.57	0.33 ± 0.12	0.18 ± 0.16	0.31 ± 0.16
	3Month		2.73 ± 0.91	0.66 ± 0.30	0.04 ± 0.34	0.16 ± 0.13	0.32 ± 0.09
	6Month		2.67 ± 0.92	0.63 ± 0.09	0.04 ± 0.26	0.16 ± 0.15	0.32 ± 0.12

*CCI* is the clear corneal incision group, *LTI* is the limbus tunnel incision group, *TMAS* the total Zernike root mean square, *HOA* the Zernike root mean square higher order aberration, *Z*_*3*_^*−1*^ the horizontal coma, *Z*_*3*_^*1*^ the Vertical coma, *Z*_*4*_^*0*^ the 4th order spherical aberration

### HOA of back corneal surface

**Fig. 3:**
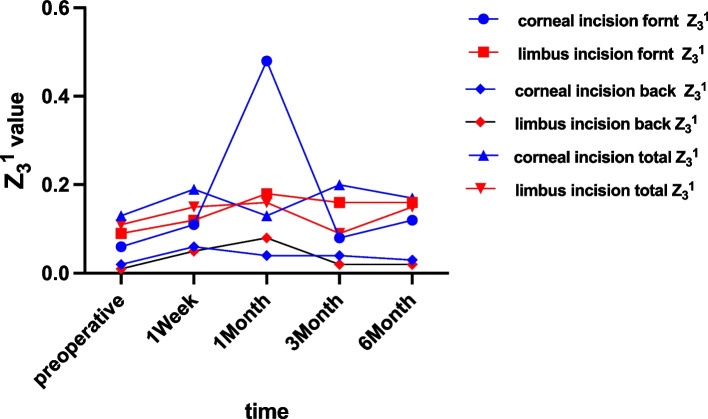
The Z_3_^1^ value of the two groups in different time

**Fig. 4:**
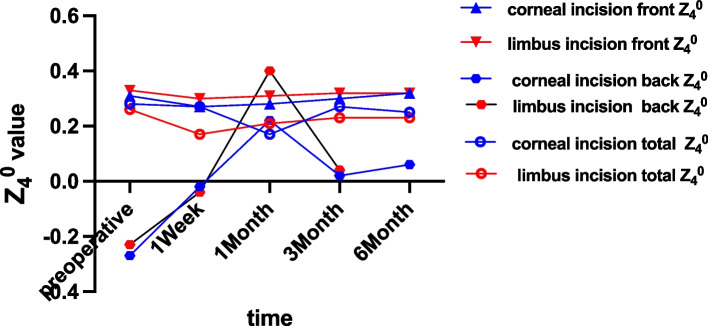
The Z_4_^0^ value of the two groups in different time

A week after the operation, the clear corneal incision group had a significantly higher Z_3_^1^ value than the limbus tunnel incision group (0.06 ± 0.06 vs. 0.05 ± 0.06, *p* = 0.031), as displayed in Fig. 3. Furthermore, the corneal back Z_4_^0^ also showed a significant difference between the clear corneal incision group and the limbus tunnel incision group (-0.02 ± 0.25 vs. -0.04 ± 0.21, *p* = 0.01), as shown in Fig. 4. Other statistics, including TMAS, HOA, Z_3_^−1^, Z_3_^1^, Z_4_^0^ revealed no significant difference between the two groups at different times (Table 3).

**Table 3:** The HOA on the back corneal surface of the two surgical incisions groups (mean ± SD)

Group	Time	n	TMAS	HOA	Z_3_^−1^	Z_3_^1^	Z_4_^0^
CCI	Preoperative	70	3.31 ± 0.27	0.30 ± 0.15	0.03 ± 0.06	0.02 ± 0.04	-0.27 ± 2.36
	1Week		1.34 ± 0.45	0.42 ± 0.36	0.01 ± 0.09	0.06 ± 0.06	-0.02 ± 0.25
	1Month		0.25 ± 0.03	0.09 ± 0.01	0.11 ± 0.02	0.04 ± 0.01	0.22 ± 0.03
	3Month		1.21 ± 0.32	0.35 ± 0.13	0.04 ± 0.05	0.04 ± 0.03	0.02 ± 0.23
	6Month		1.28 ± 0.29	0.33 ± 0.13	0.04 ± 0.06	0.03 ± 0.02	0.06 ± 0.43
LTI	Preoperative	57	4.32 ± 0.35	0.29 ± 0.17	0.04 ± 0.05	0.01 ± 0.06	-0.23 ± 0.63
	1Week		1.24 ± 0.42	0.38 ± 0.23	0.02 ± 0.07	0.05 ± 0.06^*^	-0.04 ± 0.21^*^
	1Month		0.23 ± 0.04	0.11 ± 0.02	0.09 ± 0.02	0.08 ± 0.01	0.40 ± 0.07
	3Month		1.19 ± 0.18	0.30 ± 0.06	0.05 ± 0.09	0.02 ± 0.03	-0.04 ± 0.21
	6Month		1.20 ± 0.19	0.28 ± 0.03	0.04 ± 0.04	0.02 ± 0.03	0.03 ± 0.22

*CCI* is the clear corneal incision group, *LTI* is the limbus tunnel incision group, *TMAS* the total Zernike root mean square, *HOA* the Zernike root mean square higher order, *Z*_*3*_^*−1*^ the horizontal coma, *Z*_*3*_^*1*^ the Vertical coma, *Z*_*4*_^*0*^ the 4th order spherical aberration

^*^P-value < 0.05

### HOA of total corneal

One week after the operation, the total corneal Z_4_^0^ of the clear corneal incision group was 0.27 ± 0.19, compared to 0.17 ± 0.19 in the limbus tunnel incision group (*p* = 0.32). The limbus tunnel incision group had a lower total corneal Z_4_^0^ value than the clear corneal incision group (Fig. 4). Other statistics, including TMAS, HOA, and Z_3_^−1^ showed no significant difference between the two groups at different times (Table 4).

**Table 4:** The HOA on the total corneal of the two surgical incisions groups (mean ± SD)

Group	Time	n	TMAS	HOA	Z_3_^-1^	Z_3_^1^	Z_4_^0^
CCI	Preoperative	70	2.65 ± 1.37	0.71 ± 0.49	0.20 ± 0.40	0.13 ± 0.21	0.28 ± 0.16
	1Week		3.30 ± 1.85	0.99 ± 0.66	0.18 ± 0.57	0.19 ± 0.23	0.27 ± 0.19
	1Month		2.76 ± 1.56	0.75 ± 0.64	0.22 ± 0.57	0.13 ± 0.17	0.17 ± 0.24
	3Month		2.60 ± 0.82	0.71 ± 0.368	0.04 ± 0.34	0.20 ± 0.13	0.27 ± 0.11
	6Month		2.86 ± 0.91	0.61 ± 0.169	0.05 ± 0.27	0.17 ± 0.15	0.25 ± 0.14
LTI	Preoperative		2.20 ± 0.77	0.57 ± 0.17	0.10 ± 0.25	0.11 ± 0.16	0.26 ± 0.11
	1Week	57	2.76 ± 0.97	0.85 ± 0.49	0.12 ± 0.39	0.15 ± 0.19	0.17 ± 0.19^*^
	1Month		2.60 ± 0.76	0.62 ± 0.21	0.12 ± 0.34	0.16 ± 0.18	0.21 ± 0.08
	3Month		2.22 ± 0.90	0.67 ± 0.26	0.07 ± 0.25	0.09 ± 0.17^*^	0.23 ± 0.08
	6Month		2.04 ± 0.61^*^	0.61 ± 0.11	0.14 ± 0.23	0.15 ± 0.18	0.23 ± 0.11

*CCI* is the clear corneal incision group, *LTI* is the limbus tunnel incision group, *TMAS* the total Zernike root mean square, *HOA* the Zernike root mean square higher order, *Z*_*3*_^*−1*^ the horizontal coma, *Z*_*3*_^*1*^ the Vertical coma, *Z*_*4*_^*0*^ the 4th order spherical aberration

^*^P-value < 0.05

### SIA

**Fig. 5:**
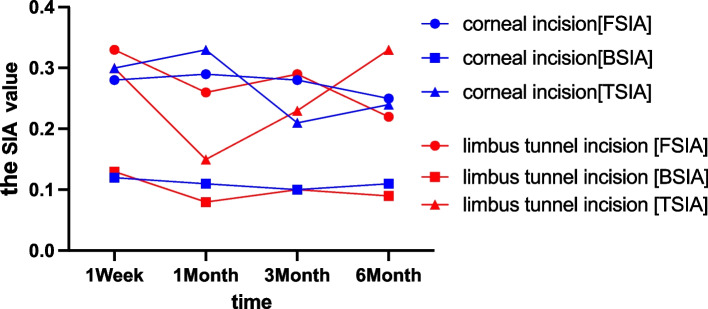
The SIA value of the two groups in different time

One week after the operation, the corneal back corneal average K value (Km) and astigmatism (CA) of the corneal incision group and the limbus tunnel incision group were (-6.42 ± 0.25, -6.39 ± 0.21, *p* = 0.02) and (0.46 ± 0.25, 0.44 ± 0.22, *p* = 0.03), respectively. The limbus tunnel incision group had higher back Km and CA values compared to the corneal incision group. One month after the operation, the corneal back SIA of the clear corneal incision group was 0.11 ± 0.11 compared to 0.08 ± 0.11 of the limbus tunnel incision group (*p* = 0.013). The clear corneal incision had a higher back SIA than the limbus tunnel incision at the one-month post-operation mark. In addition, the clear corneal incision group also had a significantly higher corneal total SIA than the limbus tunnel incision group (0.33 ± 0.30 vs. 0.15 ± 0.16, *p* = 0.004), as shown in Fig. 5.

Six months after the operation, the corneal front astigmatism and back astigmatism of the corneal incision group and the limbus tunnel incision group were (1.21 ± 0.16 vs. 1.57 ± 1.05, *p* = 0.04) and (0.38 ± 0.16 vs. 0.52 ± 0.16, *p* = 0.007), respectively. However, the total corneal astigmatism was (0.140 ± 0.52 vs. 0.83 ± 1.63, *p* = 0.410). The total SIA of the corneal incision group and the limbus tunnel incision group were (0.24 ± 0.14, 0.33 ± 0.32, *p* = 0.393). The results showed no significant difference between the two groups 6 months after the operation (Table 5).

**Table 5:** The corneal astigmatism and SIA of the two surgical incisions groups

Group	Time	n	Anterior corneal surface			Back corneal surface			Total cornal		
			Km	CA	[FSIA]	Km	CA	[BSIA]	Km	CA	[TSIA]
CCI	Preoperative		44.01 ± 0.12	1.38 ± 0.77		-6.39 ± 0.22	0.41 ± 0.18		43.21 ± 0.14	1.40 ± 0.61	
	1Week	70	43.96 ± 1.38	1.69 ± 0.81	0.28 ± 0.31	-6.42 ± 0.25	0.46 ± 0.25	0.12 ± 0.18	43.46 ± 1.12	1.40 ± 0.61	0.30 ± 0.20
	1Month		44.19 ± 1.25	1.70 ± 1.56	0.29 ± 0.29	-6.21 ± 0.03	0.16 ± 0.02	0.11 ± 0.11	43.88 ± 1.13	1.46 ± 0.64	0.33 ± 0.30
	3Month		44.11 ± 1.26	1.44 ± 0.92	0.28 ± 0.31	-6.39 ± 0.22	0.38 ± 0.15	0.10 ± 0.11	43.51 ± 1.12	1.36 ± 0.66	0.21 ± 0.12
	6Month		44.46 ± 1.28	1.21 ± 0.54	0.25 ± 0.22	-6.38 ± 0.21	0.38 ± 0.16	0.11 ± 0.13	44.26 ± 1.18	1.40 ± 0.52	0.24 ± 0.14
LTI	Preoperative		43.84 ± 1.39	1.16 ± 0.86		-6.39 ± 0.21	0.40 ± 0.17		43.34 ± 1.32	1.04 ± 0.52	
	1Week		43.79 ± 1.17	1.67 ± 0.83	0.33 ± 0.24	-6.39 ± 0.21^*^	0.44 ± 0.22^*^	0.13 ± 0.19	43.51 ± 1.38	1.04 ± 0.52	0.30 ± 0.23
	1Month	57	43.78 ± 1.15	1.87 ± 0.99	0.26 ± 0.26	-6.22 ± 0.03	0.17 ± 0.03	0.08 ± 0.10^*^	43.50 ± 1.21	1.15 ± 0.45	0.15 ± 0.16^*^
	3Month		43.87 ± 0.93	1.50 ± 1.01	0.29 ± 0.28	-6.34 ± 0.17	0.41 ± 0.19	0.10 ± 0.11	43.46 ± 0.57	0.95 ± 0.57	0.23 ± 0.10
	6Month		43.72 ± 0.79^*^	1.45 ± 1.05	0.22 ± 0.23	-6.34 ± 0.16	0.52 ± 0.17^*^	0.09 ± 0.09	43.32 ± 0.75^*^	0.83 ± 1.36	0.33 ± 0.32

*Km* the corneal average K value, *CA* the corneal astigmatism, *FSIA* the front corneal surgery astigmatism, *BSIA* the back corneal surgery astigmatism, *TSIA* the total corneal surgery astigmatism

^*^P-value < 0.05

## Discussion

ICL is generally restricted to patients with a high refractive error that is beyond the range of corneal procedures due to the risk of corneal ectasia. Precise measurements and a carefully planned procedure are essential to optimize visual quality. In recent years, the number of incisions were reduced from two to one, thereby limiting the negative impact. The limbus tunnel incision and the clear corneal incision are commonly performed. The corneal incision may be a factor to be considered for more precise calculations of the refractive index. In this study, the HOA and SIA after ICL-V4c implantation were analyzed to compare the limbus and the clear cornea incision.

Previous aberration studies revealed that the cornea is the primary source of intraocular refractive aberration. Many IOL calculations consider the impact of surgical source astigmatism SIA. Additionally, the suitability and extent of ICL surgery should also be considered. Astigmatism aberration, coma aberration, and trefoil aberration are the three main factors that affect the quality of optics. High-order aberrations include spherical aberration, coma, cloverleaf, etc.; most originate from the anterior surface of the cornea. LASIK was found to lead to more than twice the corneal spherical aberration than PIOL [15]. Phakic IOLs also induce changes in HOA due to their optic properties and the corneal incision made during the surgery [16]. CariPrez Vives’ study [17] on the effects of ICL on HOA demonstrated a negative impact on spherical aberration. Furthermore, the study conducted by Sun Woong Kim [18] examined the combined effects of ICL and corneal incision on higher-order aberrations (HOA). The results revealed that both the ICL and different large corneal incisions led to changes in spherical aberration. Torii H's research [19] demonstrated that the use of Artisan lenses resulted in a greater increase in positive spherical aberration in 6-mm pupils compared to Article lenses. As opposed to cataract surgery, small changes in the cornea can be accurately detected in ICL surgery using wavefront aberration, without the error of supergenerated energy levels. However, no study has compared the clear corneal incision and limbus tunnel incision for posterior chamber implantable collamer lens placement. The higher-order aberration of the cornea is a crucial indicator of the changes in the surgical incision following ICL surgery.

Our research reveals some differences in the corneal higher-order aberrations at different follow-up times. The main effect following ICL is the spherical aberration Z_4_^0^, showing a small change in the coma, which is in accordance with Seyed’s results [20]. However, the corneal total Z_4_^0^ of 0.27 ± 0.19 obtained in this study was higher than in Salmon and Seyed’s study [21]. Some differences in the corneal higher-order aberrations were observed at different times between the clear corneal incision group and the limbus tunnel incision group. One week after the operation, the limbus tunnel incision group demonstrated a lower corneal back Z_3_^1^, Z_4_^0^, and Km compared to the clear corneal incision group. These findings may be related to the size of the tunnel incision in the posterior cornea in relation to the central apex of the cornea. These changes gradually return to preoperative levels over 6 months by corneal self-repair. The magnitude of the phase difference was correlated with the diameter. Previous studies on ICL aberrations have shown no effect on corneal higher-order phase differences when measuring diameters of 3 mm, but the higher-order aberrations changed when measuring diameters of 6 mm. To better observe corneal changes, the diameter was adjusted to 6.5 mm. Therefore, our phase contrast results were higher than other previously published articles, and the differences were more pronounced [21].

In Phaco + IOL cataract surgery, corneal incisions flatten the perpendicular corneal meridian, resulting in surgically induced astigmatism (SIA). The magnitude of SIA depends on the size, location, and design of the incision. Previous studies have compared the value of SIA with various incision sizes, and some have also assessed its long-term evolution [22].

The limbal relaxing incisions may result in a median surgically induced astigmatism (SIA) of 1.8D-2.2D [23]. The astigmatism derived from clear keratotomy after cataract surgery starts to stabilize 2 weeks after the procedure and shows no significant change for up to 1 year or even 10 years [24, 25]. The current study found that astigmatism and aberration changes are a dynamic process, with the magnitude of change stabilizing at 6 months after the surgery. Six months after the operation, the limbus tunnel incision group had more back astigmatism and less front astigmatism, but there was no difference in total astigmatism. The total SIA of the clear corneal incision group and the limbus tunnel incision group after the operation 6 months was 0.24 ± 0.14 and 0.33 ± 0.32, respectively (*p* = 0.393); these statistics showed no significant differences between the two groups across different times, with less corneal surgery astigmatism and HOA changes after the operation 6 months.

In cataract surgery, a 3.0 mm incision may result in 0.6 D SIA [26]. In our study, both incisions caused astigmatism of less than 0.35D, and the magnitude of the changes stabilized 6 months after the procedure. The mean corneal power of total keratometry reduced slightly after cataract surgery (-0.05 dpt), which was mostly attributed to a decrease in back surface power (-0.04 dpt) [27]. The incision width and axial direction caused various changes in astigmatism of surgical origin [28]. In this study, the location of the incision was fixed in both axial direction (110) and width (3.0 mm), and the main difference between the two kinds of incision groups was the distance from the incision’s inner mouth to the central point of the cornea. The Pentacam Scheimpfug principle can directly measure both anterior and posterior corneal curvatures [29]. A gentle tunnel incision produces a longer and wider corneal section, whereas a steeper corneal incision is closer to the pupil area and scar healing leads to corneal aberrations. These changes plateau six months after the operation and have little impact on the patient’s visual outcome.

## Conclusion

In ICL-V4c implantation, the clear corneal incision and the limbus tunnel incision both affect the corneal back Z_3_^1^ and the back and total corneal Z_4_^0^ within a week after surgery. Moreover, the back and total corneal SIA are affected within a month after surgery. However, the high-order aberrations recovered to the preoperative levels 6 months after the operation in both incision groups, and the total SIA was less than 0.35D. Six months after ICL-V4c implantation, no significant difference in high-order aberration and SIA was found between the clear corneal incision and the limbus tunnel incision.

## Data Availability

The datasets generated during and/or analyzed during the current study are available from the corresponding author on reasonable request.

## Abbreviations

ICL/TICL: Implantable Contact Lens / Toric Implantable Contact Lens
RMS: Root Mean Square of aberrations
HOA RMS: Higher-order aberration
Coma RMS: Coma coefficients
SIA: Surgically induced astigmatism
CCI: Clear corneal incision group
LTI: Limbus tunnel incision group
AL: Axis length
UDVA: Uncorrected distance visual acuity
CDVA: Corrected distance visual acuity
TMAS: Total Zernike root mean Square of aberrations
HOA: Higher order Zernike root mean square
Z_3_^−^^1^: Horizontal coma
Z_3_^1^: Vertical coma
Z_4_^0^: The 4th order spherical aberration
